# Keratorefractive Effect of High Intensity Focused Ultrasound Keratoplasty on Rabbit Eyes

**DOI:** 10.1155/2016/5260531

**Published:** 2016-06-13

**Authors:** Zhiyu Du, Pisong Yan, Qiang Luo, Dan Zhang, Yu Zhang

**Affiliations:** ^1^Department of Ophthalmology, The Second Affiliated Hospital, Chongqing Medical University, Chongqing 400010, China; ^2^Medal Eye Institute, Chongqing 400050, China; ^3^Key Laboratory of Molecular Biology for Infectious Diseases (Ministry of Education), Institute for Viral Hepatitis, Department of Infectious Diseases, The Second Affiliated Hospital, Chongqing Medical University, Chongqing 400010, China; ^4^Department of Ophthalmology, Armed Police Hospital of Chongqing, Chongqing 400061, China

## Abstract

*Purpose*. To evaluate high intensity focused ultrasound (HIFU) as an innovation and noninvasive technique to correct presbyopia by altering corneal curvature in the rabbit eye.* Methods*. Eighteen enucleated rabbit eyes were treated with a prototype HIFU keratoplasty. According to the therapy power, these eyes were divided three groups: group 1 (1 W), group 2 (2 W), and group 3 (3 W). The change in corneal power was quantified by a Sirius Scheimpflug camera. Light microscopy (LM) and transmission electron microscopy (TEM) were performed to determine the effect on the corneal stroma.* Results*. In the treated eyes, the corneal curvature increases from 49.42 ± 0.30 diopters (D) and 48.00 ± 1.95 D before procedure to 51.37 ± 1.11 D and 57.00 ± 1.84 D after HIFU keratoplasty application in groups 1 and 3, respectively. The major axis and minor axis of the focal region got longer when the powers of the HIFU got increased; the difference was statistically significant (*p* < 0.05). LM and TEM showed HIFU-induced shrinkage of corneal stromal collagen with little disturbance to the underlying epithelium.* Conclusions*. We have preliminarily exploited HIFU to establish a new technique for correcting presbyopia. HIFU keratoplasty will be a good application prospect for treating presbyopia.

## 1. Introduction

Presbyopia is a progressive loss of crystalline lens accommodation that results in an inability to focus at near vision, which occurs with aging. The aging process affects each individual beginning near 40 years of age [1]. The estimated global prevalence of presbyopia was 1.04 billion people in 2005 [2]. The large number of potential patients who might seek permanent spectacle-free correction of this condition have stimulated the increasing interest in refractive surgery. In recent years, various surgical solutions [3–8] have been developed to effectively treat this condition. However, none has emerged as the final and optimum solution for presbyopia because of complications, invasiveness, and lack of predictability and stability. Thus, it is necessary to seek noninvasive, safe, and effective technique.

High intensity focused ultrasound (HIFU) is a noninvasive or minimally invasive medical procedure which has shown considerable potential for a variety of therapeutic applications [9]. Therapeutic ultrasound, although less well known than ultrasound for diagnostic imaging, has become a topic of growing interest in ophthalmology [10]. In recent years, several experimental and clinical studies for the treatment of glaucoma have been conducted [10–15]. Although several studies have addressed the effects of HIFU on the cornea in the 1980s and 1990s [16, 17], nowadays HIFU has more advantages, such as controlled accuracy, accuracy of positioning in the treatment area, and smaller focal zones [11–13]. Furthermore, a higher operating frequency allows for a steeper transition between the focal zone and the untreated area, thus reducing the risk of heating the neighboring healthy tissue [12].

Therefore, the potential therapeutic applications of HIFU might encompass many present uses of laser therapy in ophthalmology and also offer new methods of treating ocular problems. The primary goal of the present study was to establish a new technique for correcting presbyopia. So, we evaluate the histologic characteristics of corneal stroma lesions induced by HIFU in animals. We also report the change in dioptric power on rabbit eyes in vitro after HIFU corneal circular treatment.

## 2. Material and Methods

### 2.1. Experimental Animals

An approval of animal use was granted by the Animal Care and Use Committee at Chongqing Medical University. Whole animal management followed the ARVO Statement for the Use of Animals in Ophthalmic and Visual Research (ARVO Animal Policy). Enucleated eyes from adult New Zealand White rabbits (5-6 months old, 2–2.5 kg) were provided by Laboratory Animal Center of Chongqing Medical University. Rabbits of different sex were included randomly. All rabbits were sacrificed using venous air embolism before ophthalmectomy. Before all animals were sacrificed using venous air embolism, they were anaesthetized using Sumianxin II intramuscular injection (0.1 mL/kg) and all efforts were made to minimize suffering. The fat and muscle were removed from all eyes with scissors after enucleation. Within 1 hour of enucleation, the eyes were stored in isotonic saline on ice until used, not later than 24 hours after enucleation. Eighteen eyes were used.

### 2.2. Experimental Apparatus

**Figure 1:**
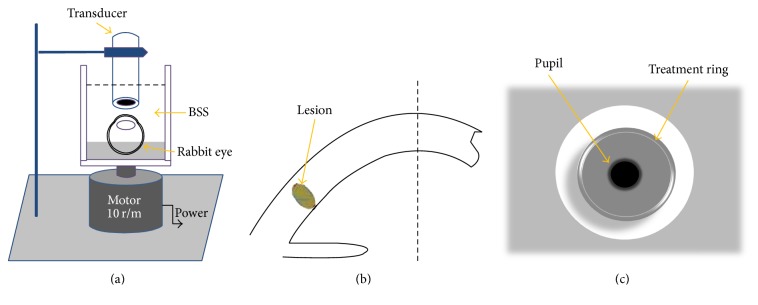
(a) Schematic diagram of HIFU keratoplasty transducer applied to an in vitro rabbit cornea cross section. (b) Cross-sectional view of the lesion of HIFU keratoplasty. (c) End view of the treatment ring.

HIFU keratoplasty treatments were delivered with a prototype, which was designed by Chongqing HIFU Technology Co, Ltd., Chongqing, China. Continuous therapeutic energy was emitted from a focal ultrasound transducer (focal length, 5 mm; focal zone, 65 *μ*m) with a working frequency of 10.2 MHz, and therapy power was set at 1 W (group 1, 6 eyes), 2 W (group 2, 6 eyes), and 3 W (group 3, 6 eyes) for an exposure time of 6 seconds. Intact rabbit eye globes were placed in a transparent Plexiglas observation cell, which was put on the rotary motor with uniform velocity (Figure 1(a)). The cell was full of balanced salt solution (BSS). The ultrasound transducer placed perpendicular to the corneal surface and both had no direct contact with each other. The focal length of the transducer was adjusted by Vernier caliper to focus on the same depth of rabbit corneal stroma. The time of running one circle of the rotary motor was also 6 seconds. A stromal treatment ring (8 mm) was produced within the peripheral corneal stroma after HIFU radiation (Figures 1(b) and 1(c)).

### 2.3. Corneal Topography

The Sirius Scheimpflug camera (CSO, Firenze, Italy) was used for corneal topographic imaging and characterized the treatment effects of the eye before and after HIFU keratoplasty application immediately. The rabbit eye was fixed with a fixed bracket, and it positioned in the front of the Sirius Scheimpflug camera. Three measurements were taken before and after HIFU keratoplasty application. BSS was applied to the cornea before all Scheimpflug measurements. The simulated keratoscope readings (Sim-K) in central 3 mm zone of the rabbit cornea were analyzed.

### 2.4. Histologic Examination

For histologic examination, the corneas were fixed in 4% buffered formaldehyde, dehydrated in alcohol solutions of increasing concentration, cleared in xylene, embedded in paraffin, and sectioned into 5 *μ*m thick sections. The sections were stained with hematoxylin-eosin and examined under a light microscope (LM). Digimizer software (version 3.1.1.0) was used for measuring the maximum width and length of the focal region.

### 2.5. Transmission Electron Microscopy (TEM)

For TEM examination, the corneas were placed into a glutaraldehyde 2.5% in 0.1 mol/L cacodylate buffer (pH 7.3) at 4°C, for at least 24 hours, and then postfixed in 1% osmium tetroxide in 0.1 mol/L cacodylate buffer (pH 7.3) at 4°C for 1 hour. After dehydration and embedding, samples were sectioned and examined under a TEM.

## 3. Results

### 3.1. Corneal Topography

**Figure 2:**
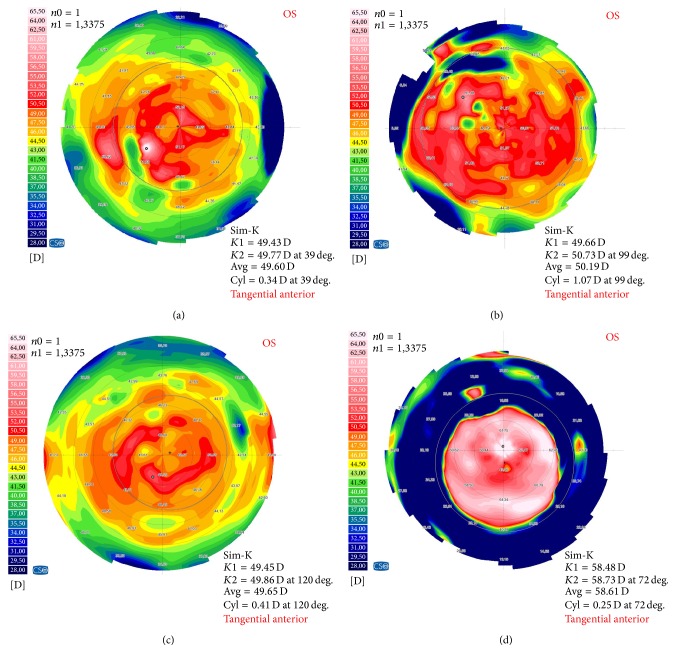
Topography display of treated cornea (Sirius, CSO, Firenze, Italy). (a) Group 1 (therapy power, 1 W). (c) Group 3 (therapy power, 3 W). Tangential curvature of the anterior corneal surface before HIFU treatment. (b, d) Tangential curvature of the same cornea after HIFU treatment. It shows steepening in the central cornea and flattening outside. Sim-K: simulated keratoscope readings,* K*1: the curvature in the flat meridian,* K*2: the curvature in the steep meridian, Avg: the mean curvature, Cyl = cylinder, deg.: degree, D: diopter, OS: left eye, and tangential anterior: tangential curvature of the anterior corneal surface.

Group 1 (1 W) and group 3 (3 W) were measured before and after HIFU keratoplasty application by using the Sirius Scheimpflug camera. The mean keratometric power (Sim-K value) for corneas before treatment was 49.42 ± 0.30 D and 48.00 ± 1.95 D in groups 1 and 3, respectively. The mean keratometric power (Sim-K value) after treatment was 51.37 ± 1.11 D and 57.00 ± 1.84 D in groups 1 and 3, respectively.  Figure 2 shows the corneal topographic variation in the tangential corneal curvature induced by HIFU treatment in a rabbit eye.

### 3.2. Histology and Electron Microscopy

**Figure 3:**
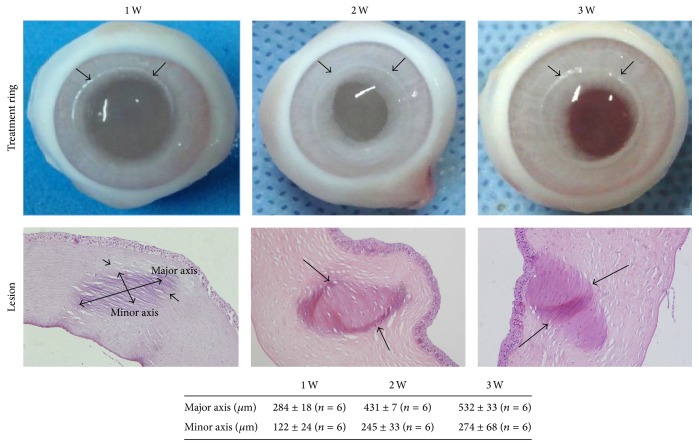
Photograph display of treatment ring in the rabbit cornea after HIFU keratoplasty treatment and photomicrograph of a rabbit cornea cross section after HIFU treatment. The loose stromal area (lesion) corresponds to a region of HIFU-induced collagen shrinkage, ×200. The table displays the major and minor axes of the oblique elliptical lesion.

As shown in Figure 3, the stroma cornea was mostly affected by HIFU keratoplasty. The area of alterations had been similar to the oblique elliptical shape in cornea. The mean major axis of the oblique elliptical shape in cornea of group 1 was 284 ± 18 *μ*m, 431 ± 7 *μ*m in group 2, and 532 ± 33 *μ*m in group 3. The mean minor axis of the oblique elliptical shape in cornea of group 1 was 122 ± 24 *μ*m, 245 ± 33 *μ*m in group 2, and 274 ± 68 *μ*m in group 3. Meanwhile, the major axis and minor axis of the focal region got longer when the powers of the HIFU got increased; the difference was statistically significant (*p* < 0.05).

**Figure 4:**
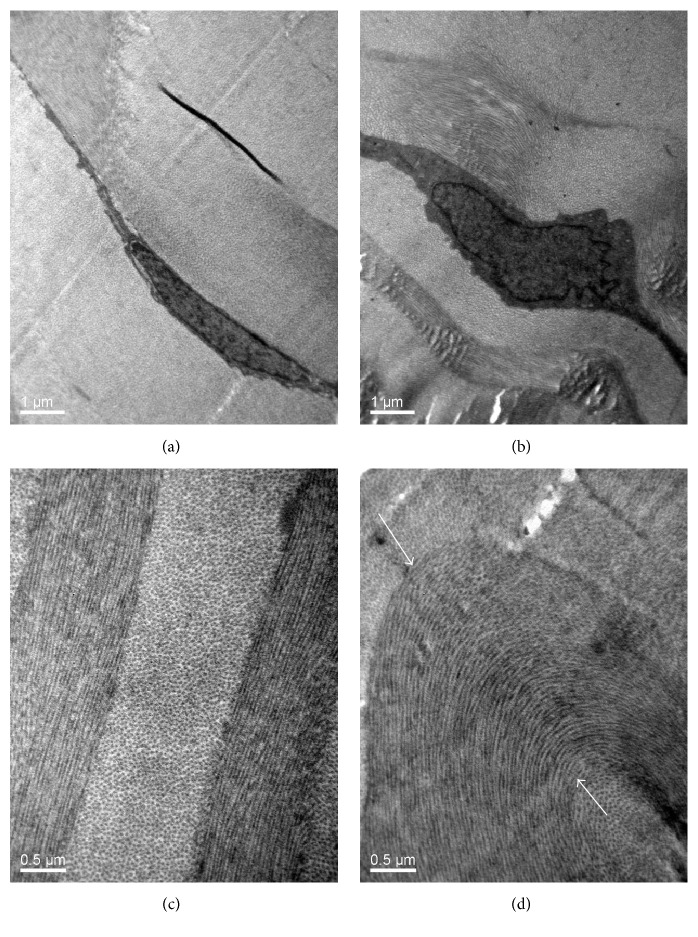
(a, c) Blank control group. (b) TEM of HIFU-treated zones demonstrated that the morphologic appearance of the keratocytes was close to normal. (d) The typical structures of stromal collagen shrinkage (white arrows) are within the HIFU treatment zone.

TEM of HIFU-treated zones demonstrated that the morphologic appearance of the keratocytes was close to normal (Figure 4(b)). Within the HIFU-affected area, the keratocytes were situated between the crumpled collagen layers, like “sandwich.” Thus the morphology and the structure of keratocytes would be affected by the crumpled collagen layers. According to Figure 4(d), the collagen layers crumpled significantly within the HIFU treatment area. Besides the typical structure of stromal collagen, microfibrillar aggregations can also be observed throughout the area of crumpled collagen layers.

## 4. Discussion

In the present study, the histologic changes and the variation in dioptric power induced by HIFU treatment in rabbit corneas were evaluated. Since 1898, using heat to change the morphology of the cornea has been employed for different therapeutic and surgical objectives. The best known of these techniques is probably thermokeratoplasty (TKP), which is based on the principle that heating corneal tissue causes collagen fibers to shrink and hence changes the corneal curvature [18]. To date, 3 main TKP technologies have emerged: laser thermokeratoplasty (LTK), conductive keratoplasty (CK), and microwave keratoplasty. However, due to complications, invasiveness, and lack of predictability and stability [19], none has emerged as the optimum solution for presbyopia. Thus, it is necessary to seek noninvasive, safe, and effective technique.

HIFU thermal treatment is a novel, minimally invasive medical procedure which has shown considerable potential for a variety of therapeutic applications [9]. The conception of HIFU was firstly brought forward by Lynn and Putnam in 1944 [20]. In 1960, W. J. Fry and F. J. Fry [21] applied HIFU technology to experimentally treat nervous system diseases and suggested the potential of HIFU in surgical operations. In ophthalmology, HIFU for the treatment of glaucoma and ultrasonic drug delivery are the two main areas of research and potential clinical application [10]. Nevertheless, HIFU keratoplasty was first described in 1990 by Rutzen et al. [17] as a theoretical option for inducing collagen shrinkage in corneas. They commented that HIFU could be used to heat the peripheral cornea with great precision because of the small focal zone and the excellent aiming capabilities of HIFU. However, with the development of high-frequency miniaturized transducer, HIFU enables the creation of smaller focal zones (65 *μ*m) that better target the treatment areas, particularly for small organs such as clear cornea. The higher operating frequency (10.2 MHz) also allows for a steeper transition between the focal zone and the untreated area. In addition, HIFU also enables a defined and adjustable tissue volume to be heated and treated at any depth or location within the eye [10]. Therefore, our group evaluated the histologic characteristics of corneal stroma lesions induced by HIFU in rabbits and also report the change in dioptric power of the procedure.

In the present study, our results demonstrated that HIFU keratoplasty increased the power of the rabbit corneas from 49.42 ± 0.30 D to 51.37 ± 1.11 D in group 1 and from 48.00 ± 1.95 D to 57.00 ± 1.84 D in group 3. The results indicated that the variations of curvature of the rabbit corneas got steeper when the powers of the HIFU got increased. Like other thermal techniques (i.e., LTK [3], CK [4]), HIFU thermal treatment is applied in the periphery of the cornea, it causes peripheral flattening and corresponding steepening in the center (Figures 2(b) and 2(d)). Moreover, compared with CK or LTK, HIFU keratoplasty showed comparable refractive results. In addition, our results show a protective effect of epithelium and basement membrane within the HIFU-treated area (Figure 3). Therefore, it is likely that this procedure would not cause postoperative pain. Moreover, as this study showed, because of the epithelium intactness, the topography examination is possible immediately after surgery. In the clinic, this would allow evaluation of clinical results immediately after the treatment. It is well known that postoperative dry eye can be commonly seen after excimer laser-based refractive surgery. The reason may be the fact that corneal nerves are cut during the procedure. However, HIFU thermal treatment requires no cutting of the cornea; therefore, the problem of postoperative dry eye and infection would not likely occur.

Our findings demonstrated that HIFU thermal treatment induced annular elliptical treatment zone in the anterior stroma (Figure 3). Although the shape of HIFU thermal lesion differs from CK, which creates a thermal footprint that is uniformly cylindrical, the effect seems to be similar to CK [22]. A full circular elliptical treatment ring of HIFU keratoplasty applied to the peripheral cornea produces a cinching effect that increases the curvature of the central cornea [23]. The stability of the induced changes is a critical point in thermokeratoplasty procedures. For this reason, further studies are necessary to determine whether there is regression of treatment effect. Our long-term goal is to use ultrasound to produce permanent alterations in corneal curvature. In addition, the time and dose of the effect on collagen fibers as well as matrix changes will have to be fully elucidated in in vivo experiment. Our group will continue to develop HIFU keratoplasty to improve repeatability and will compare its performance to existing methods for correcting presbyopia. The development of treatment parameters will be necessary to determine the optimum depth, diameter of treatment ring, and power of treatment to produce specific refractive changes.

In conclusion, the results in our study suggest that HIFU as a noninvasive method for applying in the peripheral cornea could steepen the central cornea. We have initially exploited HIFU to establish a new technique for changing the refractive index of the intermediate cornea to correct presbyopia. There will be a good application prospect for HIFU keratoplasty in the treatment of presbyopia.
